# Antagonism of the Prokineticin System Prevents and Reverses Allodynia and Inflammation in a Mouse Model of Diabetes

**DOI:** 10.1371/journal.pone.0146259

**Published:** 2016-01-05

**Authors:** Mara Castelli, Giada Amodeo, Lucia Negri, Roberta Lattanzi, Daniela Maftei, Cecilia Gotti, Francesco Pistillo, Valentina Onnis, Cenzo Congu, Alberto E. Panerai, Paola Sacerdote, Silvia Franchi

**Affiliations:** 1 Dipartimento di Scienze Farmacologiche e Biomolecolari, Università degli Studi di Milano, Milano, Italy; 2 Department of Physiology and Pharmacology ‘Vittorio Erspamer’, University of Rome, Roma, Italy; 3 Consiglio Nazionale delle Ricerche, Institute of Neuroscience, Milano, Italy; 4 Department of Life and Environmental Sciences, Unit of Pharmaceutical, Pharmacological and Nutraceutical Sciences, University of Cagliari, Cagliari, Italy; University of Kentucky Medical Center, UNITED STATES

## Abstract

Neuropathic pain is a severe diabetes complication and its treatment is not satisfactory. It is associated with neuroinflammation-related events that participate in pain generation and chronicization. Prokineticins are a new family of chemokines that has emerged as critical players in immune system, inflammation and pain. We investigated the role of prokineticins and their receptors as modulators of neuropathic pain and inflammatory responses in experimental diabetes. In streptozotocin-induced-diabetes in mice, the time course expression of prokineticin and its receptors was evaluated in spinal cord and sciatic nerves, and correlated with mechanical allodynia. Spinal cord and sciatic nerve pro- and anti-inflammatory cytokines were measured as protein and mRNA, and spinal cord GluR subunits expression studied. The effect of preventive and therapeutic treatment with the prokineticin receptor antagonist PC1 on behavioural and biochemical parameters was evaluated. Peripheral immune activation was assessed measuring macrophage and T-helper cytokine production. An up-regulation of the Prokineticin system was present in spinal cord and nerves of diabetic mice, and correlated with allodynia. Therapeutic PC1 reversed allodynia while preventive treatment blocked its development. PC1 normalized prokineticin levels and prevented the up-regulation of GluN2B subunits in the spinal cord. The antagonist restored the pro-/anti-inflammatory cytokine balance altered in spinal cord and nerves and also reduced peripheral immune system activation in diabetic mice, decreasing macrophage proinflammatory cytokines and the T-helper 1 phenotype. The prokineticin system contributes to altered sensitivity in diabetic neuropathy and its inhibition blocked both allodynia and inflammatory events underlying disease.

## Introduction

Although the number of available agents to manage diabetes continues to rapidly expand, the treatment of diabetes complications remains a substantial challenge.

Diabetic neuropathy is one of the most frequent complications in diabetes mellitus [1]. Patients with diabetic neuropathy experience different forms of paraesthesia, hyperalgesia and allodynia [2]. Many drugs are used for treating neuropathic pain (NP), but their effectiveness is limited because of insufficient efficacy and side effects [3,4]. Recently, we and others have demonstrated that pro- and anti-inflammatory cytokines produced by immune cells as well as by glia and microglia in nerve and spinal cord are common denominators in NP [5,6]. These start a cascade of neuroinflammation-related events that may maintain and worsen the original injury, participating in pain generation and chronicization [7–9]. The recently identified prokineticin system, which belongs to a new family of chemokines, has been recognized as a regulator at cross roads of inflammation and NP. The prokineticin 2 (PROK2 or mammalian Bv8), displays a major role in triggering inflammatory pain acting on two G-protein coupled receptors, the prokineticin receptor 1 (PKR_1_) and the prokineticin receptor 2 (PKR_2_) [10,11] localized in regions of the nervous system related with pain, both on neurons and glia [12]. PROK2 is also an important modulator of immune responses. Immune cells express PKRs and PROK2 exerts chemotactic activities, induces a proinflammatory macrophage phenotype and skews the Th1/Th2 balance to Th1 [13–15]. We have recently demonstrated that the block of this system with a specific receptor antagonist provides an efficacious control of development and maintenance of inflammatory pain and of NP derived from traumatic nerve lesion [12,16,17].

Considering that prokineticins and their receptors are involved in nociception, immunoregulation and inflammation, they appear as candidates for controlling pain, inflammation and neuroinflammation in diabetes. The objectives of the present study are to identify the role of PKs system in the streptozotocin model of diabetes neuropathy in mouse, investigating its involvement in allodynia, in neuronal alterations measuring NMDA receptors in spinal cord as well as by evaluating cytokine levels in the main tissue stations involved in nociception transmission and in peripheral immune responses. The results obtained suggest that the pharmacological modulation of the prokineticin system may be an innovative approach to control diabetes complications.

## Material and Methods

### Animals and Treatments

All animal care and experimental procedures complied with the International Association for the Study of Pain and European Community (E.C.L358/118/12/86) guidelines and were approved by the Animal Care and Use Committee of the Italian Ministry of Health (Permission 21/2014). All efforts were made to minimize animal suffering and to reduce the number of animals used.

Studies involving animals are reported in accordance with the ARRIVE guidelines for reporting experiments involving animals [18]. A total of 140 animals were used in the experiments described here.

C57BL/6J male mice weighing 20–25 g, 9 weeks old (Harlan Laboratories, Italy) were housed with light/dark cycles of 12 hours, temperature of 22±2°C, humidity of 55±10%, food and water ad libitum.

Diabetes was induced in mice by intraperitoneal (i.p.) administration of Moderate Low Doses of streptozotocin (MLD-STZ) (80 mg/ kg once daily for three consecutive days) [19] (Sigma Aldrich, Italy), in citrate buffer 0.1 M, pH 4.55. Control mice were injected with vehicle (citrate buffer).

Tail-vein blood glucose concentration was assessed using a glucometer (GLUCOCARD G+ meter, Menarini diagnostics, Italy). Animals with blood glucose values above 250 mg/dl were considered diabetic.

The PKR antagonist PC1, a triazine-guanidine compound [20], was dissolved in sterile saline and used at the dose of 150 μg /kg [21].

Different treatment protocols were applied.

1. The effect of a single bolus of PC1 was studied in MLD-STZ mice 21 days after diabetes induction evaluating the responses to mechanical stimuli 30, 60, 120 and 240 minutes after PC1.
2. Therapeutic protocol: animals were subcutaneously injected with PC1 or saline twice-daily for 14 consecutive days starting 21 days after MLD-STZ, when they were already hyperglycaemic and neuropathic.
3. Preventive protocol: animals were treated with PC1 or saline at the same time of STZ (day 0), i.e. when hyperglycaemia and mechanical allodynia had not yet developed.

### Mechanical Allodynia

Mechanical allodynia was monitored evaluating the mechanical touch sensitivity through a blunt probe (Von-Frey filament, 0.5 mm diameter) on the mid plantar surface of the hind paw, using the Dynamic Plantar Aesthesiometer (Ugo Basile, Italy) [22]. Responses to mechanical stimuli (paw withdrawal thresholds, PWT) were measured before neuropathy induction (T0), and weekly after MLD-STZ on both hind paws by researchers who were blind to treatments. In all experiments, in order to avoid the evaluation of potential acute effect of the antagonist on nociceptive thresholds, behavioural testing was performed 12–15 hours after the last drug administration and the following PC1 administration was immediately performed after the pain behaviour assessment.

### Tissue Sampling and Preparation

Mice were killed by CO_2_ inhalation for spinal cord (L4-L6), sciatic nerves and pancreas dissection. Tissues were immediately frozen in liquid nitrogen and conserved at -80°C until use. Spleens and peritoneal macrophages were also collected.

In all the experiments, last drug administration was performed in the evening, and tissues collected the following morning, i.e. 12–15 hours after the last PC1 administration.

### Measurement of IL-1β and IL-10 in Sciatic Nerve, Spinal Cord and Pancreas

Sciatic nerves and spinal cord samples were homogenized in 0.3 ml of ice-cold phosphate-buffered saline containing a protease inhibitor cocktail (lysis buffer) (Roche Diagnostics, Italy). Pancreatic tissues were homogenized in 2 ml of the same lysis buffer. Samples were centrifuged at 1000 g for 15 min at 4°C and supernatants collected to measure cytokines levels and total protein content (Lowry’s method).

### Measurement of GluR subunits in Spinal Cord

Nine mice/group were used for these experiments. Spinal cords from 3 mice belonging to the same treatment groups were pooled, and the experiments repeated twice.

Samples were homogenized using a potter in Tris-HCl buffer (50mM Tris, 120 mM NaCl, 5mM KCl, 2.5 mM, 1mM MgCl2, pH 7), washed once by centrifugation (1h; 25000g) and then resuspended in the proper volume of the same buffer containing a 10 μg/ ml mixture of each of the following protease inhibitors: leupeptin, bestatin, pepstatin A and aprotinin.

Total protein content was evaluated by using BCA protein assay (Pierce Chemical, Rockford, IL).

### Cell Sampling and Stimulation for Cytokine Assay

#### Macrophages

Macrophage collection was performed at the end of therapeutic PC1 treatment 35 days after MLD-STZ injections. At this time point mice were killed and peritoneal cells (PECs) were harvested in RPMI 1640 medium (Sigma-Aldrich) plus 10% FCS. Cell viability was checked by the Trypan blue exclusion test. Turk solution was used to discriminate nuclei and on the basis of their morphology, cells were counted.

The cells obtained from mice belonging to the same treatment groups were pooled.

PECs were diluted in collection medium at the final concentration of 1x10^6^/ml, and 1 ml/well aliquots were dispensed into 24-well culture plates.

Isolation and purification of macrophages were carried out by 2 hours of adherence to plates. As previously reported [23], this procedure produces a population of macrophages with a 90% purity. Non-adherent cells were removed, and adherent cells washed twice with PBS solution and incubated with or without 1 μg/ ml LPS for IL-1β and IL-10 stimulation. The stimulus was added to the macrophage cultures in a final volume of 1 ml/well in RPMI 1640 plus 10% FCS, 1% glutamine, 2% streptomycin solution and 0,1% 2-mercaptoethanol (complete RPMI).

After 24 hours of culture at 37°C in 5% CO_2_ and 95% air, the supernatant was collected and stored frozen at -80°C for cytokine evaluation.

#### Splenocytes

Spleen cell collection was performed at the end of therapeutic PC1 treatment 35 days after MLD-STZ injections.

At this time point mice were killed and their spleens rapidly and aseptically removed. Splenocytes were spilled out from an incision on spleen cuticle made with 20-gauge needles, adjusted in 24-well plates at the final concentration of 4x10^6^ cell/ ml of culture medium (complete RPMI, i.e. RPMI 1640 supplemented with 10% FCS, 1% glutamine, 2% antibiotics and 0.1% 2-mercaptoethanol) and incubated at 37°C in 5% CO_2_ and 95% air with or without 10 μg/ ml Concanavalin A (ConA) for Th1 and Th2 cytokine stimulation. The stimulus was added to the cell cultures in a final volume of 1 ml/well in complete RPMI.

After 24 (in the case of IFN-γ and IL-2) or 48 hours (in the case of IL-4 and IL-10) of culture, times of maximum cytokines release [23–25], the supernatant was collected and stored frozen at -80°C for cytokine assay.

In order to evaluate the T-helper (Th)1/Th2 balance, the IFN-γ/IL-4 ratio was calculated for each mouse and calculated by setting IFN-γ/IL-4 ratio of the control as 100%.

### Real Time PCR

Total RNA was isolated from sciatic nerves and the lumbar spinal cords using Trizol® reagent (Trifast Eurogold®, Euroclone, Italy) according to manufacturer’s instructions and re-suspended in 10–20 μl of RNases-free water. All procedures were performed as previously described in details [12,22]. Specific TaqMan probe/primers for mouse Prokineticin 2 (Prok2 Mm 01182450_g1), Prokineticin receptors (Prokr1 Mm00517546_m1; Prokr2 Mm00769571_m1), interleukins (IL-1β Mm00434228_m1; IL-10 Mm00439616_m1) and glyceraldehydes-3-phosphate dehydrogenase (GAPDH Mm99999915_g1) were purchased from Applied Biosystems. Threshold cycle numbers (Ct) of the specific gene of interest and the endogenous control gene GAPDH were determined by ABI PRISM 7000 Sequence Detection System.

The Ct value of the specific gene of interest was normalized to the Ct value of the endogenous control, GAPDH, and the comparative Ct method (2^−ΔΔCt^) was then applied using control/ non diabetic group as calibrator.

### ELISA

Cytokine concentration was determined using ultra-sensitive ELISA kits according to the manufacturer’s instruction. DuoSet® ELISA development system s for mouse IL-1β, IL-2, IFN-γ and IL-4 were purchased from R&D Systems (Minneapolis, USA) while mouse IL-10 ELISA Ready-SET-Go from eBioscience (San Diego, CA). Cytokine concentrations were reported as pg cytokine/ mg total protein content in spinal cord, sciatic nerve and pancreas. Cytokine production by macrophages and splenocytes was reported as concentrations in culture media of stimulated cultures.

Plasma Insulin levels were determined using a specific ELISA kit for mouse (Mercodia, Uppsala, Sweden)

### Western Blot

#### Antibody production and characterization

We used affinity-purified, subunit-specific polyclonal antibodies (Abs), produced in rabbit against peptides derived from the C-terminal (COOH), N-terminal (NH) of mouse and AMPAR GluA1 and GluA2/3 subunits. The Ab against the GluA2/3 subunit was directed against the C-terminus peptide (EGYNVYGIESVKI). The Ab against the GluA1 subunit was directed against the extracellular domain peptides (RTSDSRDHTRVDWKR) corresponding to aminoacids 253–267 (271–285 if numbered from the signal peptide), this region is not conserved in GluA2-4, nor Kainate and NMDAR. GluA1 and GluA2/3 sequences were the same as those reported by Chemicon International. The specificity of the affinity-purified Abs was previously tested by western blotting studies using cells transfected and non-transfected with GluA1 and GluA2/3. Our tests do not show crossreactivity between GluA1 and GluA2/3 Abs, as it has been reported in the specificity tests of Chemicon International.

Anti GluN1 (clone 54.1) was from BD Pharmigen, anti GluN2A (clone A3-2D10) was from Invitrogen, anti-GluN2B (clone N59/20) was from Antibodies Incorporated, anti-tubulin (clone B-5-1-2) was from Sigma-Aldrich and anti Na/K ATPase was described in [26].

#### Immunoblotting and densitometric quantification of Western blot bands

The analysis of the GluR subunits by Western blotting was performed as described previously [27]. In brief, depending on the target subunit, 2.5, 5, or 10 μg of total homogenates samples were diluted 1:1 (v/v) with Laemmli buffer and then underwent SDS-PAGE using 7.5% acrylamide gel. After SDS-PAGE, the proteins were electrophoretically transferred to nitrocellulose membranes with 0.45-mm pores (Schleicher and Schull, Dassel, Germany). The blots were blocked overnight in 4% non-fat milk in Tris-buffered saline, washed in a buffer containing 4% nonfat milk and 0.3% Tween 20 in Tris-buffered saline, incubated for 2 h with the primary antibody at the following concentration (GluA1 and GluA2/3: 1–2.5 mg /ml; GluN1 1:1500; GluN2A 1:1000; GluN2B 1:600; Na/K ATPase 1:1000; tubulin 1:10000) and then incubated for 1h with the appropriate secondary antibody (anti-rabbit Ly-CorIRDye800RD: 1:10000; anti-mouse Ly-Cor IRDye680RD: 1:7500). Membranes were further washed in Tris-buffered saline and dried overnight at RT in darkness. The IR signal was measured through the IR scanner Odyssey CL220x - Infrared Imaging System.

The quantification of the signal intensity of the Western blot bands was performed with iStudio software. The optical density ratio was calculated by taking the optical density of the control as 100%. The values are the mean ± SEM of 3 replications for each antibody, performed on 3 separate pools.

### Data Analysis

Results are presented as means ± SEM of 6–8 animals/group. Statistical analyses were performed using one-way or two-way ANOVA for parametric results. Follow-up analysis was performed using the Tukey’s test or Bonferroni’s post tests for multiple comparisons, respectively. T Student test was used for the comparison between two groups. In the case of non-parametric results, Kruskal-Wallis ANOVA was applied, followed by Dunn’s test.

All the statistical analysis was performed using GraphPad Prism 5 Software (San Diego, CA, U.S.A) Differences were considered significant at p < 0.05.

## Results

### Time course of hyperglycemia, mechanical allodynia and PROK2 mRNA levels in spinal cord after MLD-STZ

**Fig 1:**
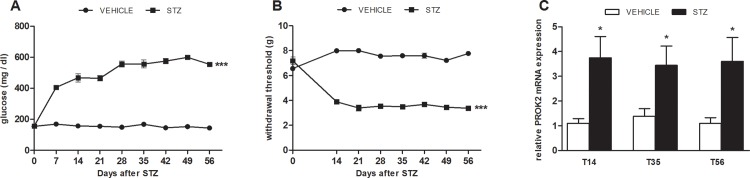
Parallel development of mechanical allodynia and increase of spinal cord PROK2. Time-course of hyperglycemia (A) and mechanical allodynia (B) after MLD-STZ (80 mg /kg for 3 consecutive days). Data represent mean± SEM of 6 mice per group. Two way ANOVA was used for statistical evaluation, followed by Bonferroni’s test. ***p<0.001 vs vehicle/CTR. C: time-course of mRNA expression levels of PROK2 in spinal cord (L4-L6) of mice injected with MLD-STZ. The mRNA levels, determined by Real Time PCR, were expressed in relation to GAPDH and presented as fold-increases over the levels in vehicle/CTR animals. Data represent mean ±SEM of 6 mice per group. Student T Test was used for statistical evaluation *p<0.05, vs vehicle/CTR.

As indicated in Fig 1, MLD-STZ induces in animals a rapid hyperglycemic state (panel A) associated to the development of allodynia (panel B). On day 14 after MLD-STZ the PWT of diabetic mice were reduced compared to controls and remained significantly lower for the entire period of observation (B).

As depicted in panel C an increase of PROK2 mRNA expression appeared in the spinal cord of diabetic mice 14 days after the MLD-STZ, corresponding to clear manifestation of allodynic symptoms and was still significant at day 56.

### Modulation of allodynia

**Fig 2:**
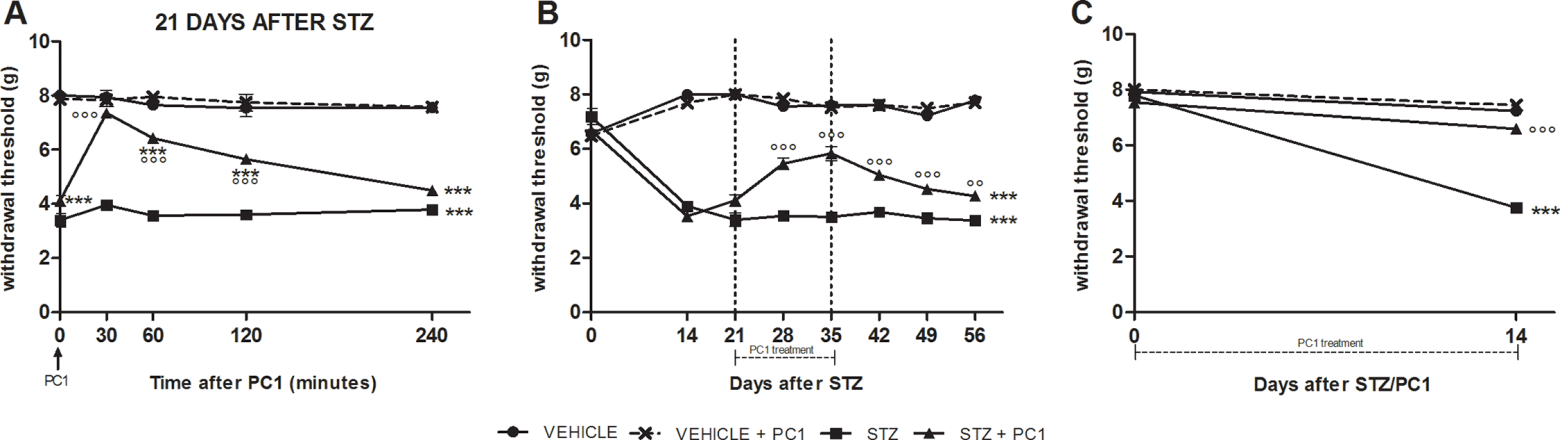
Anti-allodynic effect of PKR antagonist (PC1) administration. Anti-allodynic effect of PC1 administration as a single bolus (A) or as repeated administrations (B, C). A: acute PC1(s.c. 150 μg/ kg) was administered 21 days after MLD-STZ. B: therapeutic PC1 protocol- PC1 was administered (s.c. 150 μg/ kg, twice-daily) for 14 days, from day 21 to 35 after MLD-STZ. C: preventive PC1 treatment-PC1 was administered (s.c. 150 μg /kg, twice-daily) for 14 days, starting from day 0, time point corresponding to the first STZ administration. Data represent mean± SEM of 6 mice / group. Two way ANOVA was used for statistical evaluation, followed by Bonferroni’s test. ***p<0.001 vs vehicle/CTR and CTR + PC1; °°p<0.01, °°°p<0.001 vs STZ.

A single bolus of PC1 on day 21 after MLD-STZ produced a total recovery in the decreased PWT of MLD-STZ mice abolishing the established mechanical allodynia in 30 minutes. The anti-allodynic effect lasted for about 2 hours and gradually disappeared within 4 hours after PC1 (Fig 2A).

The therapeutic administrations of PC1 starting 21 days after STZ for two weeks (i.e. until 35 days after MLD-STZ), was able to effectively alleviate mechanical allodynia. As shown in Fig 2B the PWT of STZ/PC1 mice were already significantly higher than PWT of STZ/saline mice after 7 day of treatment and the effect was even more pronounced at the end of 14 day treatment. Moreover after PC1 suspension the re-appearance of allodynia was delayed: the PWT remained significantly elevated although progressively decreasing (Fig 2B).

When the PK antagonist was administered in the preventive protocol together with STZ for two weeks, it significantly prevented the development of allodynia in MLD-STZ mice, as depicted in Fig 2C.

Since, as reported in Fig 2, PC1 administration in normal animals did never modify PWT in order to reduce the number of animals, this group was not included in the subsequent experiments.

### Effect of PC1 administration on body weight, glucose and insulin blood levels

**Fig 3:**
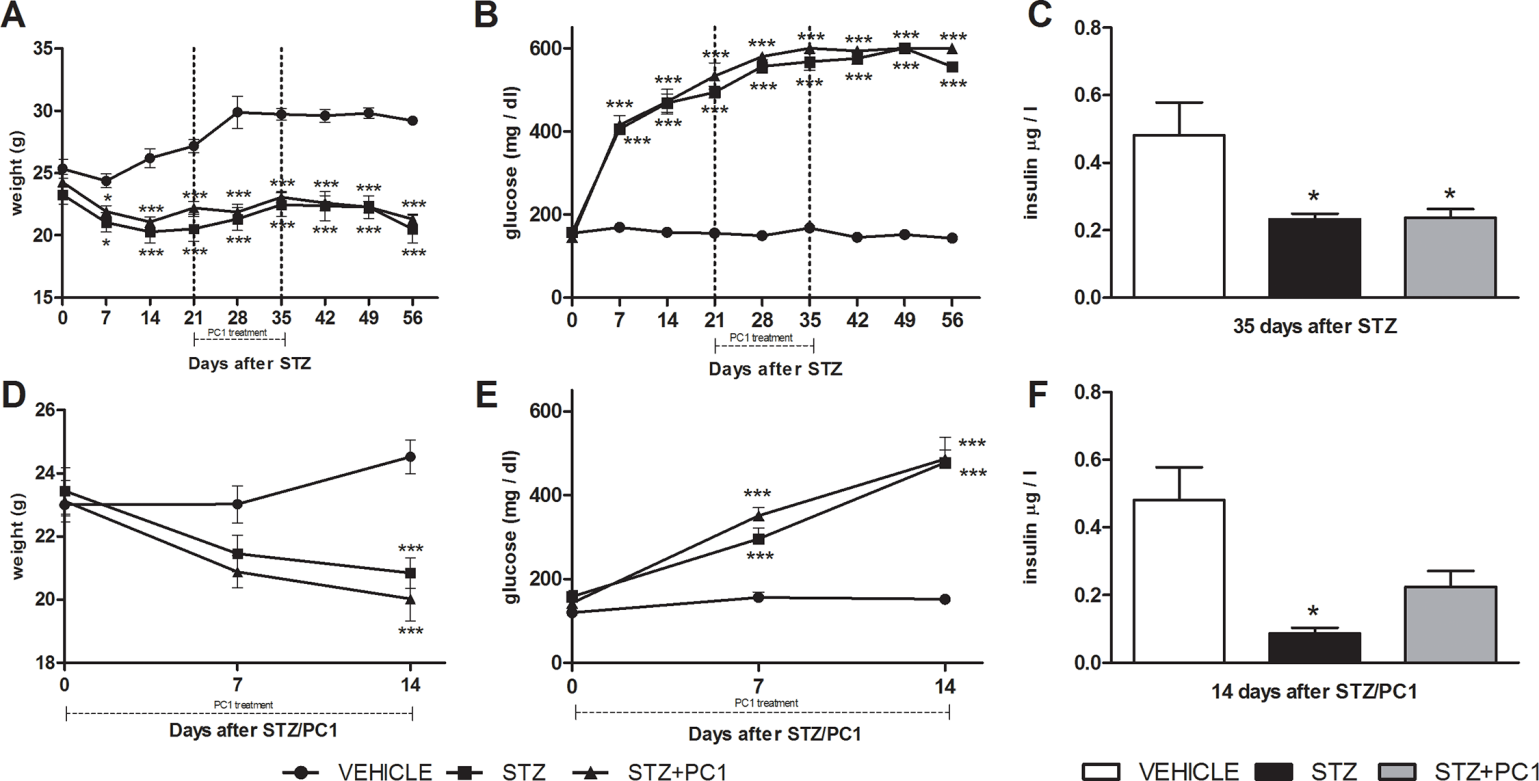
PC1 does not affect body weight, blood glucose and insulin levels. Effect of PC1 administration from day 21 to day 35 on body weight (panel A), blood glucose levels (panel B) and insulin levels (panel C). Plasma insulin levels were measured at the end of the PC1 therapeutic treatment, i.e. on day 35 after MLD-STZ. Effect of preventive PC1 administration (from day 0 to 14) on body weight (panel D), blood glucose levels (panel E) and insulin levels (panel F). Plasma insulin levels were measured at the end of the PC1 therapeutic treatment, i.e. on day 14 after MLD-STZ. ***p<0.001 vs vehicle; * p<0.05 vs vehicle

In respect to non-diabetic control animals, a significant body weight loss was observed in diabetic mice, and neither therapeutic (Fig 3, panel A) nor preventive (Fig 3, panel D) PC1 treatment did modulate it. Similarly, either therapeutic (panel B) or preventive (panel E) PC1 treatments were not able to modify blood glucose levels in MLD-STZ mice. We also measured insulin blood levels at the end of a 14 days therapeutic (panel C) or preventive (panel F) treatment with PC1: no significant modulation by the antagonist was ever present.

### Effect of PKR antagonist on PROK2, PKR_1_ and PKR_2_ mRNA expression in spinal cord and sciatic nerve

**Fig 4:**
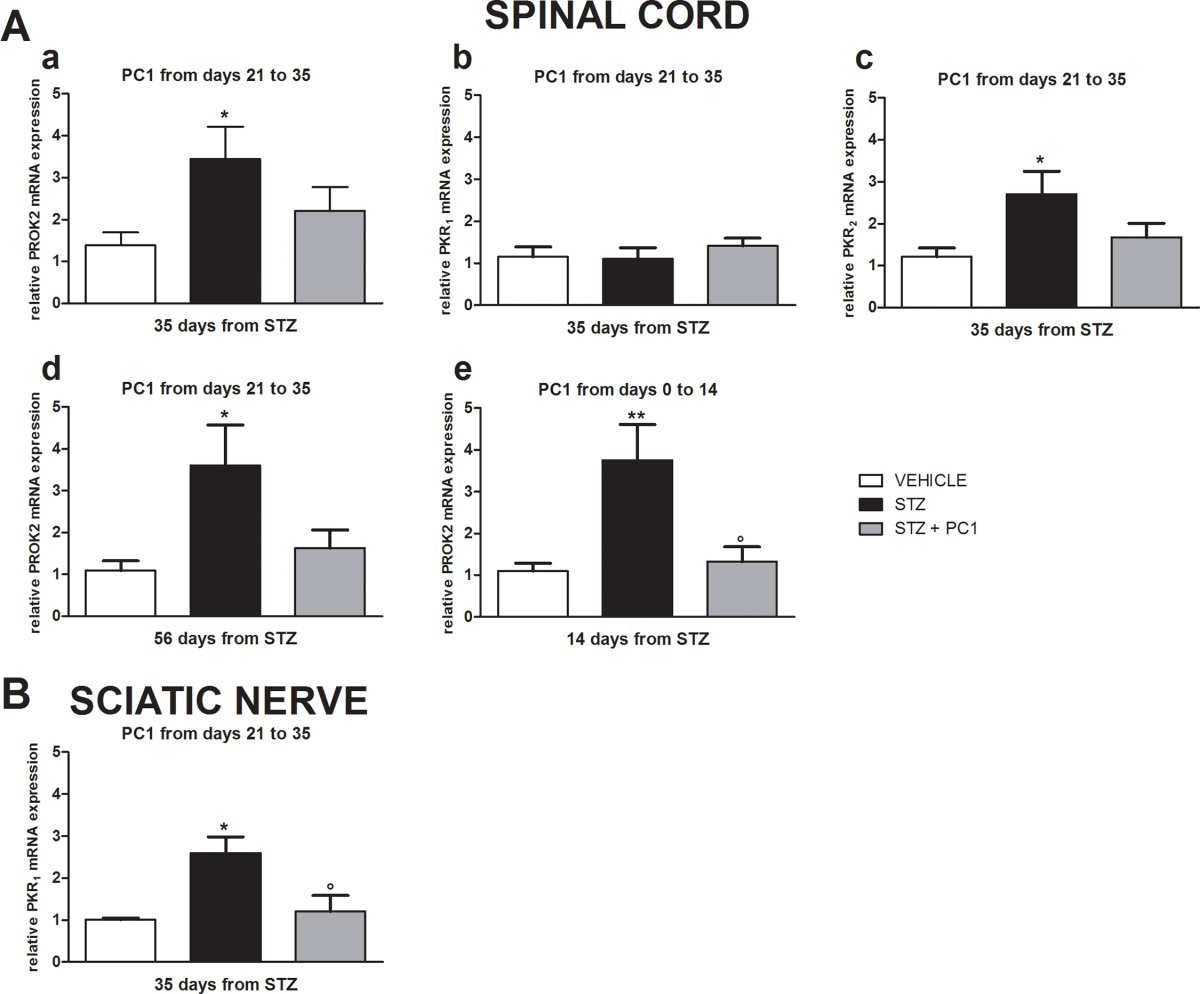
PROK2 and PKRs are overexpressed in spinal cord and sciatic nerve in diabetic mice, and the PKR antagonist PC1 normalizes them. (A) PROK2, PKR1, PKR2 mRNA expression in spinal cord (L4-L6), (a)(b)(c) 35 days after diabetes induction with MLD-STZ, at the end of therapeutic PC1 treatment (S.C. 150 μg/ kg, twice-daily from day 21 to 35 after MLD-STZ). (d) PROK2 mRNA 56 days after diabetes induction with MLD-STZ, 21 days after the discontinuation of the therapeutic PC1 treatment; (e)PROK2 mRNA expression 14 days after the initiation of preventive PC1 treatment (s.c. 150 μg/ kg, twice-daily for 14 days, starting from day 0, time point corresponding to the first STZ administration). B) PROK2 mRNA expression in sciatic nerve 35 days after diabetes induction with MLD-STZ at the end of therapeutic PC1 treatment (s.c. 150 μg/ kg, twice-daily from day 21 to 35 after MLD-STZ). The mRNA levels, determined by Real Time PCR, were expressed in relation to GAPDH and presented as fold-increases over the levels in CTR animals. Data represent mean± SEM of 6 mice per group. One way ANOVA was used for statistical evaluation, followed by Tukey’s test for multiple comparisons. *p<0.05, ** p<0.01 vs vehicle, °p<0.05 vs STZ.

Thirty five days after MLD-STZ, the PROK2 (Fig 4A, a) and PKR_2_ mRNA levels (A, c) were higher in spinal cord of diabetic mice than in controls, whereas no significant difference of PKR_1_ expression (A, b) was evident.

The PROK2 (panel a) and PKR_2_ (panel c) up-regulation were significantly reduced by a therapeutic 14 day PC1 treatment that started on day 21 after diabetes induction. Interestingly the spinal cord PROK2 levels were still reduced 21 days after PC1 discontinuation, corresponding to 56 days after MLD-STZ (panel d).

In the preventive protocol, PC1 was administered for 14 days starting at the same moment of diabetes induction. As reported in panel e, also in this condition PC1 significantly contrasted PROK2 upregulation.

Thirty five days after MLD-STZ a significant increase of PKR_1_ mRNA levels was observed also in sciatic nerve (Fig 4B). Therapeutic PC1 administrations were effective in reducing PKR_1_ up-regulation to basal in the sciatic nerve. Real-Time PCR analysis for PROK2 and PKR_2_ mRNA evaluation failed to determine their expression levels in the sciatic nerve (data not shown).

### Spinal cord glutamate receptor modulation by diabetes and preventive PC1 treatment

**Fig 5:**
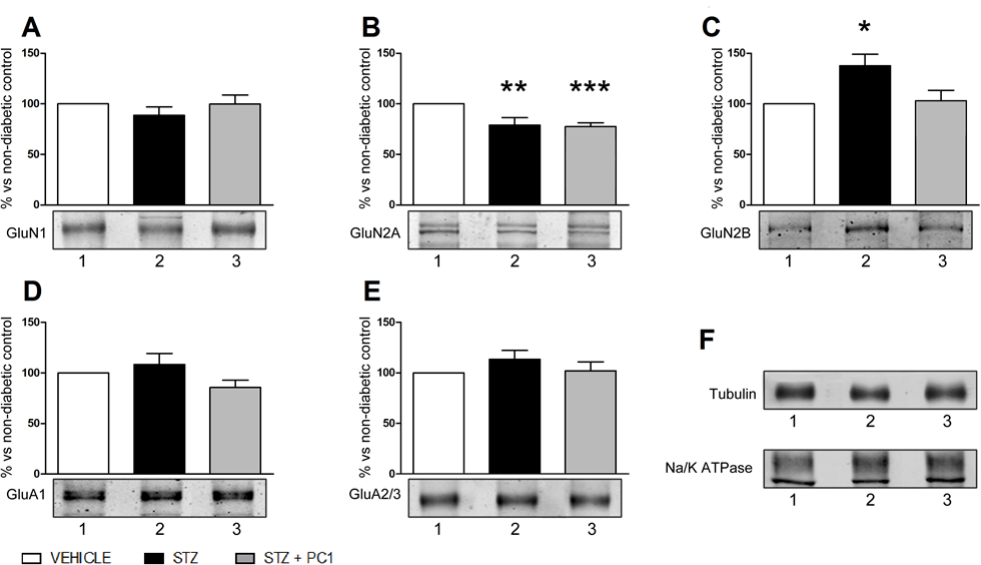
PC1 administration prevents GluN2B overexpression in spinal cord. Effect of preventive PC1 administrations (14 days) on GluN1 (A), GluN2A (B), GluN2B (C), GluA1 (D) and GluA2/3 (E) subunit content and representative Western blot bands in spinal cord 14 days after MLD-STZ. Levels of glutamate receptor subunits were normalized on tubulin. Na/K ATPase was used as controls for the integrity of plasma membrane proteins (F). The values are the mean ± SEM of 3 replications for each antibody, performed on 3 separate pools. *p<0.05,**p<0.01 ***p<0.001 vs vehicle.

As reported in Fig 5, 14 days after MLD-STZ a decrease of the NMDA receptor subunit GluN2A was present (B), while the expression of the GluN2B subunit significantly increased (C). Early PC1 administration was effective in preventing GluN2B up-regulation in spinal cord (C), but did not affect the GluN2A subunit reduction (B). As further control we also checked for the possible effect of treatments on the expression of other GluR subunits, but we didn’t find any change in the expression of AMPA receptors containing GluA1 and GluA2/3 subunits and in the level of the NMDA receptors containing the GluN1 subunit neither after MLD-STZ nor after preventive PC1 treatment (A, D and E).

### Effect of therapeutic PKR blocking on cytokine levels in spinal cord and sciatic nerve

**Fig 6:**
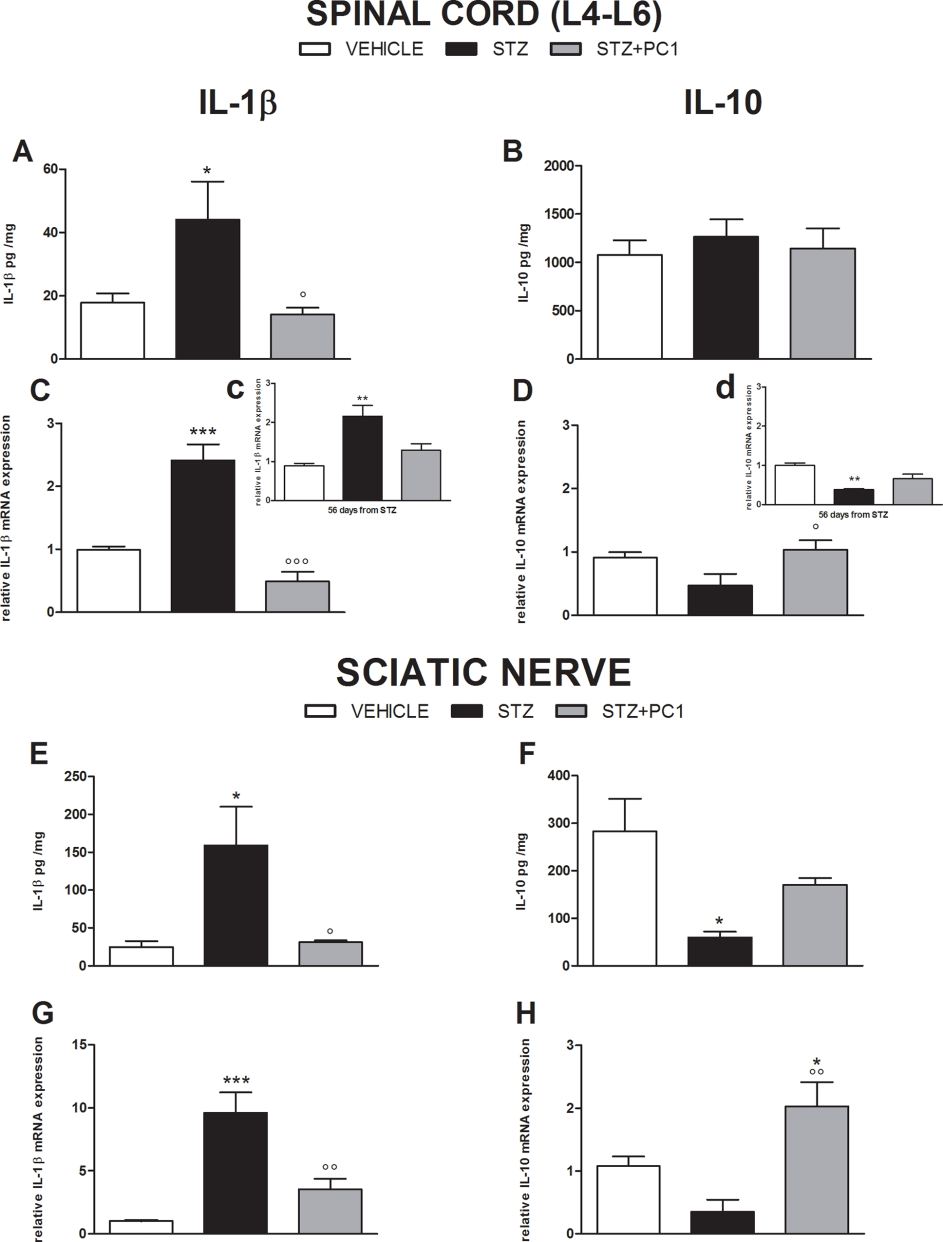
PKR antagonist PC1 restores a correct pro-anti-inflammatory cytokine balance in spinal cord and sciatic nerve. Effect of therapeutic PC1 administrations on cytokines levels in spinal cord and sciatic nerve. IL-1β (A, C, E and G) and IL-10 (B, D, F and H) protein content and mRNA expression in spinal cord and sciatic nerve 35 days after diabetes induction with MLD-STZ, at the end of therapeutic treatment with PC1 (s.c. 150 μg/ kg, twice-daily for 14 days starting on day 21 from the first STZ injection). Cytokine protein content, evaluated by ELISA, was reported as pg cytokine/ mg total proteins (A, B, E and F). Cytokine mRNA levels, determined by Real Time PCR, were expressed in relation to GAPDH and presented as fold-increase over the levels in vehicle/CTR animals (C, D, G and H). Inset c and d: expression levels of IL-1β (inset c) and IL-10 (inset d) evaluated 56 days after STZ, corresponding to 3 weeks after last PC1 injection. Data represent mean ±SEM of 6–8 mice per group. One way ANOVA was used for statistical evaluation, followed by Tukey’s test for multiple comparisons. *p<0.05, ** p<0.01, ***p<0.001 vs vehicle; °p<0.05, °°p<0.01, °°°p<0.001 vs STZ.

As illustrated in Fig 6, 35 days after MLD-STZ, protein and mRNA levels of IL-1β were increased in spinal cord (A and C) and in the sciatic nerve (E and G) of diabetic mice. Therapeutic PC1 administrations from day 21 to 35, efficaciously contrasted IL-1β up-regulation both in spinal cord and sciatic nerve.

In spinal cord no modulation of IL-10 protein levels was observed (B). On the contrary, IL-10 mRNA expression was slightly decreased in MLD-STZ mice and PC1 normalized it (D).

When we evaluated spinal cord IL-1β and IL-10 mRNA 56 days after MLD-STZ, corresponding to 3 weeks after PC1 last treatment, the effect of treatment was still present, although less evident. In fact both IL-1β (panelC, inset c) and IL-10 (panel D, inset d) levels in PC1 treated MLD-STZ animals were still not different from those of non diabetic mice, although they were not anymore significantly different from MLD-STZ.

In sciatic nerve of MLD-STZ mice the protein content and the mRNA of IL-10 were decreased compared to controls (F and H). As shown in panel H, PC1 administrations led to IL-10 mRNA production above the physiological levels and this contrasted the decrease of IL-10 protein (F) induced by MLD-STZ.

### Effect of PKR antagonism on peripheral macrophage and splenocyte cytokines

**Fig 7:**
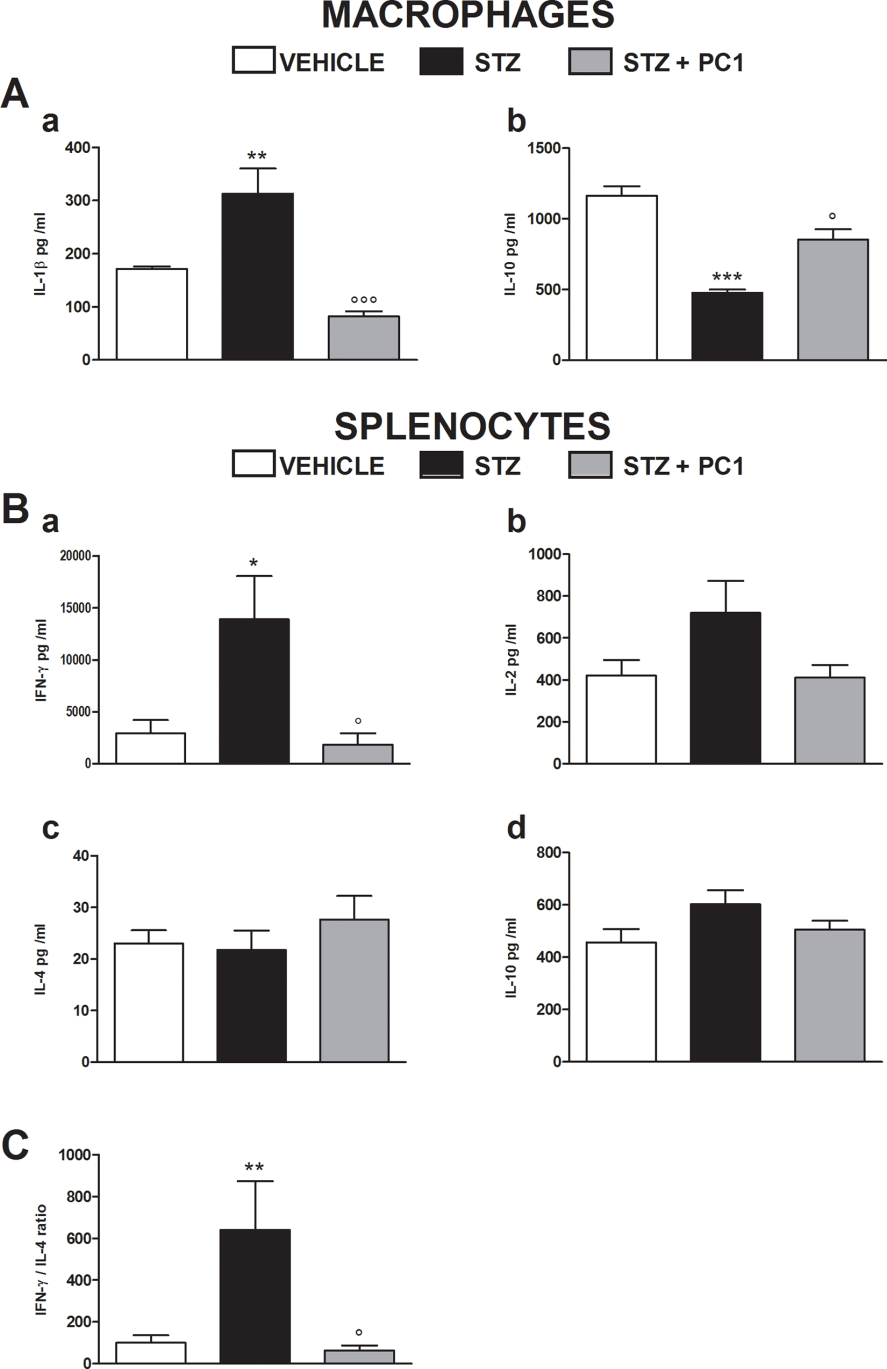
Effects of therapeutic PC1 administrations on cytokine production by macrophage and splenocytes. (A) Effect of therapeutic PC1 administrations (s.c. 150 μg/ kg, twice-daily for 14 days from day 21 to 35 after MLD-STZ) on IL-1β (a) and IL-10 (b) production by peritoneal macrophages. Data represent mean± SEM of 6–8 mice per group. (B) Effect of therapeutic PC1 administrations (s.c. 150 μg/ kg, twice-daily for 14 days from day 21 to 35 after MLD-STZ) on IFN-γ (a), IL-2 (b), IL-4 (c) and IL-10 (d) production by splenocytes. (C) Splenocyte Th1/Th2 profile was expressed as IFN-γ/IL-4 ratio and calculated by setting IFN-γ/IL-4 ratio of the control as 100%. Cytokine content, evaluated by ELISA, was reported as protein concentrations in culture media. Data represent mean ±SEM of 6 mice per group. One way ANOVA was used for statistical evaluation, followed by Tukey’s test for multiple comparisons. *p<0.05 vs vehicle/ CTR; °p<0.05 vs STZ.

Thirty five days after MLD-STZ, an alteration of peripheral cytokines was observed. A significant increase of IL-1 β production by MLD-STZ macrophages was present, while IL-10 levels were decreased (Fig 7A, a and b, respectively). PC1 administrations (therapeutic treatment) were effective in reverting these alterations, re-establishing cytokine balance. In order to assess the status of the Thelper cytokine balance, we decided to measure the more representative and prototype cytokines for Th1, i.e. IFN-γ and IL-2, and for Th2, i.e.IL-4 [24]. Moreover we also evaluated IL-10 production, since this cytokine was shown to be very sensible to PROK2 regulation [15].

In splenocytes, we observed a marked increase of IFN-γ levels (Fig 7B, a) and, even if not significant, higher concentration of IL-2 (Fig 7B, b). A protective effect induced by PC1 treatment (from day 21 to day 35 after MLD-STZ) was strongly evident on IFN-γ production as shown in panel a. The production of IL-4 and IL-10 was never altered (7B, panels c and d) in splenocytes. IFN-γ/IL-4 ratio is frequently used as index for determining T helper profile. As shown in Fig 6C, PC1 administrations completely prevented the alteration of Th1/Th2 balance in diabetic mice, where it was significantly shifted towards Th1.

We measured cytokine levels in pancreas of diabetic animals, 35 days after the induction of the pathology, and we observed a significant decrease of IL-10 level, that was 27.53 ± 3.5 ng /mg proteins in healthy animals, and was reduced to 10.88 ± 0.64 ng/ mg proteins in diabetic mice. Therapeutic PC1 treatment prevented IL-10 decrease in the pancreatic tissue (24.33 ± 5.507 ng/ mg proteins), further sustaining the anti-inflammatory efficacy of the treatment.

## Discussion and Conclusion

In this work we provide evidences about the involvement of the recently discovered chemokine PROK2 and its receptors PKR_1_ and PKR_2_, in diabetic painful neuropathy and in the related inflammatory events. Our data demonstrate that in diabetic mice spinal cord PROK2 is implicated both in the early stage of allodynia development as well as in its maintenance. Indeed an over expression of PROK2 in spinal cord was present since the appearance and for all the persistence of allodynia.

The PKRs antagonist PC1 was highly effective in relieving diabetes-induced hypersensitivity. Repeated administrations of PC1 significantly reduced mechanical allodynia in STZ mice treated in presence of fully developed neuropathy, while it completely prevented its development when given to animals not yet neuropathic. Interestingly the therapeutic schedule delayed the reappearance of painful symptoms after PC1 suspension, suggesting that blocking PROK2 signalling could induce long lasting changes in the neuronal circuits or in the neuroinflammatory phenomena involved. Moreover, a single acute injection of PC1 rapidly reduced established allodynia suggesting a direct action on nociceptor PKRs, whose blockade decreases the transmission of painful stimuli [12].

The anti-allodynic effect observed after chronic PC1 treatment could be ascribed to its ability to prevent or reduce the availability of a potent pro-nociceptive/pro-inflammatory agent such as PROK2 itself in the spinal cord. Therapeutic PC1 administrations reduced or prevented PROK2 and PKR_2_ increase in spinal cord of STZ mice. The ability of PC1 to counteract the PROK2 overexpression seems to be mediated by the PKR_1_ as it was demonstrated a specific involvement of this receptor in regulating PROK2 levels during inflammation and in nerve lesion neuropathic models [12,17,21,28]. In normal and malignant myeloid cell lines a similar positive loop between PROK2 stimulation of PKR_1_ and STAT3-mediated PROK2 production has been described [29,30]. In addition, the prevention of PROK2 up-regulation in the spinal cord was long lasting and persisted after suspension of PC1. We previously showed in the CCI injury model that PROK2 is up-regulated in spinal cord astrocytes and primary sensory neurons [12] and it may activate the PKR_2_ expressed in spinal cord neurons and overexpressed after injury. The observation that in mice precociously treated with PC1 allodynia did not develop, led us to hypothesize that when PC1 treatment started on day 0, i.e. when the plasticity of the central nervous system that underlies allodynia had not yet established [31], the blocking of PKR_2_ and PROK2 synthesis in spinal cord may prevent or slow down this neuronal plasticity.

In order to specifically evaluate the effect of PKR antagonism on neuronal sensitisation, we evaluated its effect on glutamatergic receptors in spinal cord, that are hyperactivated in diabetic animals [32,33].

Evidences indicate that alterations in synaptic transmission are associated to NP, including phosphorylation of NMDA receptors, altered NMDA receptor subunit expression pattern and increased NMDA-mediated current [34,35]. Nociceptive hypersensitivity induced by peripheral injury or tissue inflammation is known to be mediated by the activation of GluN2B containing NMDAR distributed in spinal cord dorsal horns [36–38]. Moreover, Iwata et al. [39] demonstrated that in spinal neurons the expression of GluN2A decreased while that of GluN2B increased after peripheral nerve injury. Consistently, we demonstrate that in presence of fully developed mechanical allodynia a decrease of the spinal NMDAR subunit GluN2A was present, while the expression of the GluN2B is increased. Early PC1 administrations were effective in preventing GluN2B up-regulation in spinal cord of diabetic mice, and this effect may contribute to the observed allodynia reduction. Interestingly in an in vitro model of cerebral injury, glutamate induced PROK2 expression in cortical neurons through NMDA receptor activation [40], suggesting a positive loop between PROK2, glutamate and NMDA, that could be reduced by antagonising PKR activation with the antagonist.

An important contribution to the antiallodynic activity of PC1 may also be ascribed to the relevant role that the prokineticin system plays in modulating peripheral immune/inflammatory reactions and neuroinflammation. We demonstrated that PC1 treatment significantly reduced IL-1β overexpression while enhancing IL-10 mRNA in the spinal cord in STZ mice. As we had previously reported for the CCI model [12,16], it can be suggested that by reducing PROK2 and PKR_2_ levels in spinal cord, the signalling leading to IL-1β production is significantly blunted. We are aware that in the present work we did not characterize the cellular source of the cytokines in the spinal cord. However both microglia and astrocytes have been indicated as the main cells responsible for cytokine production [41,42]. Interestingly we previously demonstrated that both PKRs and PROK2 are extensively expressed in astrocytes, where they appear upregulated in the chronic constriction injury model [12], suggesting the relevance of these cells in prokineticin-induced cytokine modulation.

When we analysed IL-1β and IL-10 56 days after diabetic induction, they were still significantly altered, consistently with the presence of allodynia. Interestingly the effect of PC1 on the levels of these cytokines started to subside 3 weeks after its suspension but did not completely disappeared, since IL-10 levels remained upregulated in the PC1 group, IL-1β upregulation was still reduced, although at a lesser extent, and also PROK levels were still slightly decreased. At this time point a mild anti-allodynic effect is still present, indicating a possible correlation with the neuroinflammatory markers. In future experiments we plan to follow the system also for a longer time.

We are aware that other proinflammatory cytokines, such as TNF-α or IL-6, have also been involved in spinal pain modulation [6,8]. However in our preview work [5,7] we measured only a transient short lasting alteration of TNF in spinal cord of neuropathic animals, while IL-1β appeared the proinflammatory cytokines more stably overexpressed for a long period of time in spinal cord in the presence of neuropathic pain [5,7]; for this reason we focussed on this proinflammatory cytokine.

From our data, it also emerges a control of PROK2 on the balance of pro- and anti-inflammatory cytokines in the sciatic nerve. In the diabetic nerve, Schwann cells, resident and macrophages recruited from bloodstream present a clear pro-inflammatory phenotype, characterized by high IL-1β and low IL-10 levels [7,43]. The antagonism with PC1 was efficacious in reverting this pro-inflammatory phenotype. Indeed we measured extremely high levels of sciatic nerve IL-10, suggesting that the block of PKRs drives nerve macrophages towards an anti-inflammatory direction. In previous work from our group in the CCI neuropathic model, we demonstrated that although IL-1β and IL-10 are the more affected cytokines after PC1 treatment, we also observed a minor modulation of other cytokines such as TNF and IL-17 in the nerve [16]. Although we did not specifically measure them in this work, it is possible that other cytokines may be involved in the sciatic nerve.

The augmentation of PKR_1_ mRNA expression in the nerve could be attributed to infiltrating inflammatory cells expressing this receptor. Since PKR_1_ is the most implicated in the immune response and mediates macrophage chemotaxis [14], it can be assumed that its blocking with PC1 could affect macrophage migration, reducing the recruitment of further inflammatory cells expressing PKR_1_. These data are in agreement with the relevance of peripheral monocytes/macrophages recruitment recently reported in the generation of neuropathic pain in a chemotherapy-induced neuropathy model [44].

Inflammation and immune activation have been recognized as fundamental mechanisms in the pathophysiology of diabetes and of its complications [45]. An autoimmune reactivity characterized by a T helper 1 profile [45,46] is consistently present in diabetes, and elevated pro-inflammatory cytokines, such as IFN-γ, TNF-α, and IL-1β [46–48] also participate in pancreatic beta-cells destruction. In agreement with the potent immunomodulatory activity of PROK2 [14,15,49,50], we now demonstrate that PROK2 and PKRs are involved also in modulating the peripheral immune response in the STZ model. PC1 treatment reduced the peripheral inflammatory status, decreasing macrophage IL-1β and maintaining Th1/Th2 balance. Interestingly, the block of PKRs was also able to prevent IL-10 alteration in pancreas. Up to now we do not know whether this effect is mediated by PKRs that have been shown to be present on pancreatic tissues [51–53] or depends on the general peripheral immunomodulation achieved with PC1.

Therefore we hypothesize that PROK2 may be involved in the several inflammatory processes that take place in diabetes. If this hypothesis is true, the blocking of PKRs could have beneficial effects on other diabetic complications, such as retinopathy and nephropathy [54].

Although PC1 blocked the STZ-induced allodynia, neuroinflammation and peripheral immune activation, it did not affect hyperglycaemia nor plasma insulin levels. However this is not uncommon, since other compounds, such as cannabis extracts, buprenorphine and ghrelin, have beneficial action on diabetic alterations and tissue damage without affecting STZ-induced hyperglycaemia [55–57]

In conclusion, we suggest the PK system as a promising target for novel pharmacological approaches to treat diabetes complications such as painful neuropathy.

## References

[1] Aring AM, Jones DE, Falko JM. Evaluation and prevention of diabetic neuropathy. Am Fam Physician 2005; 71:2123–2128. 15952441

[2] Vinik AI, Suwanwalaikorn S, Stansberry KB, Holland MT, McNitt PM, Colen LE. Quantitative measurement of cutaneous perception in diabetic neuropathy. Muscle Nerve 1995; 18:574–584. 7753119 10.1002/mus.880180603

[3] Galer BS, Gianas A, Jensen MP. Painful diabetic polyneuropathy: epidemiology, pain description, and quality of life. Diabetes Res Clin Pract. 2000; 47:123–128. 10670912 10.1016/s0168-8227(99)00112-6

[4] Kapur D. Neuropathic pain and diabetes. Diabetes Metab Res Rev.2003; 19 Suppl 1:S9–S15. 12577253 10.1002/dmrr.359

[5] Sacerdote P, Franchi S, Moretti S, Castelli M, Procacci P, Magnaghi V, et al Cytokine modulation is necessary for efficacious treatment of experimental neuropathic pain. J Neuroimmune Pharmacol. 2013; 8:202–211. 10.1007/s11481-012-9428-2 23242694

[6] Old EA, Clark AK, Malcangio M. The role of glia in the spinal cord in neuropathic and inflammatory pain. Handb Exp Pharmacol. 2015; 227:145–70. 10.1007/978-3-662-46450-2_8 25846618

[7] Valsecchi AE, Franchi S, Panerai AE, Rossi A, Sacerdote P, Colleoni MP. The soy isoflavone genistein reverses oxidative and inflammatory state, neuropathic pain, neurotrophic and vasculature deficits in diabetes mouse model. Eur J Pharmacol. 2011; 650:694–702. 10.1016/j.ejphar.2010.10.060 21050844

[8] Sommer C, Kress M. Recent findings on how proinflammatory cytokines cause pain: peripheral mechanisms in inflammatory and neuropathic hyperalgesia. Neurosci Lett. 2004; 361:184–187. 15135924 10.1016/j.neulet.2003.12.007

[9] Austin PJ, Moalem-Taylor G. The neuro-immune balance in neuropathic pain: involvement of inflammatory immune cells, immune-like glial cells and cytokines. J Neuroimmunol. 2010; 229:26–50. 10.1016/j.jneuroim.2010.08.013 20870295

[10] Negri L, Lattanzi R. Bv8/PK2 and prokineticin receptors: a druggable pronociceptive system. Curr Opin Pharmacol. 2012; 12:62–66. 10.1016/j.coph.2011.10.023 22136937

[11] Vellani V, Colucci M, Lattanzi R, Giannini E, Negri L, Melchiorri P, et al Sensitization of transient receptor potential vanilloid 1 by the prokineticin receptor agonist Bv8. J Neurosci. 2006; 26:5109–5116. 16687502 10.1523/JNEUROSCI.3870-05.2006 PMC6674238

[12] Maftei D, Marconi V, Florenzano F, Giancotti LA, Castelli M, Moretti S, et al Controlling the activation of the Bv8/prokineticin system reduces neuroinflammation and abolishes thermal and tactile hyperalgesia in neuropathic animals. Br J Pharmacol. 2014; 171:4850–4865. 10.1111/bph.12793 24902717 PMC4294109

[13] Dorsch M, Qiu Y, Soler D, Frank N, Duong T, Goodearl A, et al PK1/EG-VEGF induces monocyte differentiation and activation. J Leukoc Biol. 2005; 78: 426–434. 15908459 10.1189/jlb.0205061

[14] Martucci C, Franchi S, Giannini E, Tian H, Melchiorri P, Negri L, Sacerdote P. Bv8, the amphibian homologue of the mammalian prokineticins, induces a proinflammatory phenotype of mouse macrophages. Br J Pharmacol. 2006; 147: 225–234. 16299550 10.1038/sj.bjp.0706467 PMC1615858

[15] Franchi S, Giannini E, Lattuada D, Lattanzi R, Tian H, Melchiorri P, et al The prokineticin receptor agonist Bv8 decreases IL-10 and IL-4 production in mice splenocytes by activating prokineticin receptor-1. BMC Immunol. 2008; 9:60 10.1186/1471-2172-9-60 18957080 PMC2584092

[16] Lattanzi R, Maftei D, Marconi V, Florenzano F, Franchi S, Borsani E, et al Prokineticin 2 Upregulation in the Peripheral Nervous System Has a Major Role in Triggering and Maintaining Neuropathic Pain in the Chronic Constriction Injury Model. Biomed Research International 2015; Article ID 301292 10.1155/2015/301292 PMC4313068 25685780

[17] Guida F, Lattanzi R, Boccella S, Maftei D, Romano R, Marconi V, et al PC1, a non-peptide PKR1-preferring antagonist, reduces pain behavior and spinal neuronal sensitization in neuropathic mice. Pharmacol Res. 2015; 91:36–46. 10.1016/j.phrs.2014.11.004 25434589

[18] McGrath J, Drummond G, McLachlan E, Kilkenny C, Wainwright C. Guidelines for reporting experiments involving animals: the ARRIVE guidelines. Br J Pharmacol. 2010: 160: 1573–1576. 10.1111/j.1476-5381.2010.00873.x 20649560 PMC2936829

[19] Noh JR, Hwang JH, Kim YH, Kim KS, Gang GT, Kim SW, et al The orphan nuclear receptor small heterodimer partner negatively regulates pancreatic beta cell survival and hyperglycemia in multiple low-dose streptozotocin-induced type 1 diabetic mice. Int J Biochem Cell Biol.2013; 45:1538–1545. 10.1016/j.biocel.2013.05.004 23680671

[20] Balboni G, Lazzari I, Trapella C, Negri L, Lattanzi R, Giannini E, et al Triazine compounds as antagonists at Bv8-prokineticin receptors. J Med Chem. 2008; 51:7635–7639. 10.1021/jm800854e 19006379

[21] Giannini E, Lattanzi R, Nicotra A, Campese AF, Grazioli P, Screpanti I, et al The chemokine Bv8/prokineticin 2 is up-regulated in inflammatory granulocytes and modulates inflammatory pain. Proc Natl Acad Sci U S A 2009; 106:14646–14651. 10.1073/pnas.0903720106 19667192 PMC2731841

[22] Martucci C, Trovato AE, Costa B, Borsani E, Franchi S, Magnaghi V, et al The purinergic antagonist PPADS reduces pain related behaviours and interleukin-1 beta, interleukin-6, iNOS and nNOS overproduction in central and peripheral nervous system after peripheral neuropathy in mice. Pain 2008; 137:81–95. 17900807 10.1016/j.pain.2007.08.017

[23] Martucci C, Franchi S, Lattuada D, Panerai AE, Sacerdote P. Differential involvement of RelB in morphine-induced modulation of chemotaxis, NO, and cytokine production in murine macrophages and lymphocytes. J Leukoc Biol. 2007; 81:344–354. 17023558 10.1189/jlb.0406237

[24] Moretti S, Castelli M, Franchi S, Raggi MA, Mercolini L, Protti M, et al Δ^9^-Tetrahydrocannabinol-induced anti-inflammatory responses in adolescent mice switch to proinflammatory in adulthood. J Leukoc Biol. 2014; 96:523–534. 10.1189/jlb.3HI0713-406RR 24744434

[25] Sacerdote P, Manfredi B, Gaspani L, Panerai AE. The opioid antagonist naloxone induces a shift from type 2 to type 1 cytokine pattern in BALB/cJ mice. Blood 2000; 95:2031–2036. 10706871

[26] Pietrini G, Matteoli M, Banker G, Caplan MJ. Isoforms of the Na,K-ATPase are present in both axons and dendrites of hippocampal neurons in culture. Proc Natl Acad Sci U S A 1992;89:8414–8418 1326755 10.1073/pnas.89.18.8414 PMC49930

[27] Gotti C, Moretti M, Meinerz NM, Clementi F, Gaimarri A, Collins AC, et al Partial deletion of the nicotinic cholinergic receptor alpha 4 or beta 2 subunit genes changes the acetylcholine sensitivity of receptor-mediated 86Rb+ efflux in cortex and thalamus and alters relative expression of alpha 4 and beta 2 subunits. Mol Pharmacol. 2008; 73:1796–1807. 10.1124/mol.108.045203 18337473

[28] Negri L, Lattanzi R Bv8-prokineticins and their receptors: modulators of pain. Curr Pharm Biotechnol. 2011; 12:1720–1727. 21466441 10.2174/138920111798357410

[29] Xin H, Lu R, Lee H, Zhang W, Zhang C, Deng J, et al G-protein-coupled receptor agonist bv8/prokineticin-2 and STAT3 protein form a feed-forward loop in both normal and malignant myeloid cells. J Biol Chem. 2013; 288:13842–13849. 10.1074/jbc.M113.450049 23548897 PMC3650420

[30] Qu X, Zhuang G, Yu L, Meng G, Ferrara N. Induction of Bv8 expression by granulocyte colony-stimulating factor in CD11b+Gr1+cells: key role of Stat3 signaling. J Biol Chem. 2012; 287:19574–19584. 10.1074/jbc.M111.326801 22528488 PMC3365993

[31] Woolf CJ. Central sensitization: implications for the diagnosis and treatment of pain. Pain 2011; 152: S2–S15. 10.1016/j.pain.2010.09.030 20961685 PMC3268359

[32] Gupta M, Singh J, Sood S, Arora B. Mechanism of antinociceptive effect of nimodipine in experimental diabetic neuropathic pain. Methods Find Exp Clin Pharmacol. 2003; 25:49–52. 12690707 10.1358/mf.2003.25.1.772547

[33] Malcangio M, Tomlinson DR. A pharmacologic analysis of mechanical hyperalgesia in streptozotocin/diabetic rats. Pain 1998; 76:151–157. 9696468 10.1016/s0304-3959(98)00037-2

[34] Guo W, Zou S, Guan Y, Ikeda T, Tal M, Dubner R, Ren K. Tyrosine phosphorylation of the NR2B subunit of the NMDA receptor in the spinal cord during the development and maintenance of inflammatory hyperalgesia. J Neurosci.2002; 22:6208–6217. 12122079 10.1523/JNEUROSCI.22-14-06208.2002 PMC6757905

[35] Karlsson U, Sjoedin J, Moeller A, Johansson S, Wikstroem L, Naesstroem J. Glutamate-induced currents reveal three functionally distinct NMDA receptor populations in rat dorsal horn-effects of peripheral nerve lesion and inflammation. Neuroscience 2002; 112:861–868. 12088745 10.1016/s0306-4522(02)00140-9

[36] Boyce S, Wyatt A, Webb JK, O'Donnell R, Mason G, Rigby M, et al Selective NMDA NR2B antagonists induce antinociception without motor dysfunction: correlation with restricted localization of NR2B subunit in dorsal horn. Neuropharmacol. 1999; 38:611–623. 10.1016/s0028-3908(98)00218-4 10340299

[37] Tan PH, Yang LC, Shih HC, Lan KC, Cheng JT. Gene knockdown with intrathecal siRNA of NMDA receptor NR2B subunit reduces formalin-induced nociception in the rat. Gene Ther. 2005; 12:59–66. 15470478 10.1038/sj.gt.3302376

[38] Taniguchi K, Shinjo K, Mizutani M, Shimada K, Ishikawa T, Menniti FS, et al Antinociceptive activity of CP-101,606, an NMDA receptor NR2B subunit antagonist. Br J Pharmacol. 1997; 122:809–812. 9384494 10.1038/sj.bjp.0701445 PMC1565002

[39] Iwata H, Takasusuki T, Yamaguchi S, Hori Y. NMDA receptor 2B subunit-mediated synaptic transmission in the superficial dorsal horn of peripheral nerve-injured neuropathic mice. Brain Res. 2007; 1135:92–101. 17198690 10.1016/j.brainres.2006.12.014

[40] Cheng MY, Lee AG, Culbertson C, Sun G, Talati RK, Manley NC et al Prokineticin 2 is an endangering mediator of cerebral ischemic injury. Proc Natl Acad Sci U S A. 2012; 109:5475–80. 10.1073/pnas.1113363109 22431614 PMC3325724

[41] Calvo M, Dawes JM, Bennett DL. The role of the immune system in the generation of neuropathic pain. Lancet Neurol. 2012; 11:629–42. 10.1016/S1474-4422(12)70134-5 22710756

[42] Clark AK, Old EA, Malcangio M. Neuropathic pain and cytokines: current perspectives. J Pain Res. 2013; 21:803–14. 10.2147/JPR.S53660 PMC3839806 24294006

[43] Skundric DS, Lisak RP. Role of neuropoietic cytokines in development and progression of diabetic polineuropathy: from glucose metabolism to neurodegeneration. Exp Diabesity Res. 2003; 4:303–312. 14668051 10.1155/EDR.2003.303 PMC2478613

[44] Old EA, Nadkarni S, Grist J, Gentry C, Bevan S, Kim KW, et al Monocytes expressing CX3CR1 orchestrate the development of vincristine-induced pain. J Clin Invest. 2014; 124:2023–36. 10.1172/JCI71389 24743146 PMC4001538

[45] Agrawal NK, Kant S. Targeting inflammation in diabetes: newer therapeutic options. World J Diabetes 2014; 5:697–710. 10.4239/wjd.v5.i5.697 25317247 PMC4138593

[46] Padgett LE, Broniowska KA, Hansen PA, Corbett JA, Tse HM. The role of reactive oxygen species and proinflammatory cytokines in type 1 diabetes pathogenesis. Ann N Y Acad Sci. 2013; 1281:16–35. 10.1111/j.1749-6632.2012.06826.x 23323860 PMC3715103

[47] Rivero A, Mora C, Muros M, Garcia J, Herrera H, Navarro-Gonzalez JF. Pathogenic perspectives for the role of inflammation in diabetic nephropathy. Clin Sci (Lond) 2009; 116:479–492. 19200057 10.1042/CS20080394

[48] Gonzalez-Clemente JM, Mauricio D, Richart C, Broch M, Caixàs A, Megia A, et al Diabetic neuropathy is associated with activation of the TNF-alpha system in subjects with type 1 diabetes mellitus. Clin Endocrinol (Oxf) 2005; 63:525–529. 16268804 10.1111/j.1365-2265.2005.02376.x

[49] LeCouter J, Zlot C, Tejada M, Peale F, Ferrara N. Bv8 and endocrine gland-derived vascular endothelial growth factor stimulate hematopoiesis and hematopoietic cell mobilization. Proc Natl Acad Sci U S A 2004 101:16813–16818. 15548611 10.1073/pnas.0407697101 PMC528996

[50] Abou-Hamdan M, Costanza M, Fontana E, Di Dario M, Musio S, Congiu C, et al Critical role for prokineticin 2 in CNS autoimmunity. Neurol Neuroimmunol Neuroinflamm. 2015; 9;2:e95. 10.1212/NXI.0000000000000095 PMC4396530 25884014

[51] Jiang X, Abiatari I, Kong B, Erkan M, De Oliveira T, Giese NA, et al Pancreatic islet and stellate cells are the main sources of endocrine gland-derived vascular endothelial growth factor/prokineticin-1 in pancreatic cancer. Pancreatology 2009; 9:165–172. 10.1159/000178888 19077468

[52] Cline GW, Zhao X, Jakowski AB, Soeller WC, Treadway JL. Islet-selectivity of G-protein coupled receptor ligands evaluated for PET imaging of pancreatic β-cell mass. Biochem Biophys Res Commun. 2011; 412:413–418. 10.1016/j.bbrc.2011.07.077 21820405

[53] Dormishian M, Turkeri G, Urayama K, Nguyen TL, Boulberdaa M, Messaddeq N, et al Prokineticin receptor-1 is a new regulator of endothelial insulin uptake and capillary formation to control insulinsensitivity and cardiovascular and kidney functions. J Am Heart Assoc. 2013: 2:e000411 10.1161/JAHA.113.000411 24152983 PMC3835255

[54] Barutta F, Bruno G, Grimaldi S, Gruden G. Inflammation in diabetic nephropathy: moving toward clinical biomarkers and targets for treatment. Endocrine. 2015; 48:730–42 10.1007/s12020-014-0437-1 25273317

[55] Comelli F, Bettoni I, Colleoni M, Giagnoni G, Costa B. Beneficial effects of a Cannabis sativa extract treatment on diabetes-induced neuropathy and oxidative stress. Phytother Res. 2009; 23:1678–1684. 10.1002/ptr.2806 19441010

[56] Canta A, Chiorazzi A, Meregalli C, Carozzi V, Oggioni N, Lauria G, et al Continuous buprenorphine delivery effect in streptozotocin-induced painful diabetic neuropathy in rats. J Pain 2009; 10:961–968. 10.1016/j.jpain.2009.04.003 19595641

[57] Kyoraku I, Shiomi K, Kangawa K, Nakazato M. Ghrelin reverses experimental diabetic neuropathy in mice. Biochem Biophys Res Commun. 2009; 389:405–408. 10.1016/j.bbrc.2009.08.171 19733151

